# Hirudin Ameliorates Renal Interstitial Fibrosis via Regulating TGF-*β*1/Smad and NF-*κ*B Signaling in UUO Rat Model

**DOI:** 10.1155/2020/7291075

**Published:** 2020-06-26

**Authors:** Kang Yang, Boya Fan, Qingyun Zhao, Yue Ji, Panying Liu, Shan Gao, Tong Ren, Yitian Dou, Ming Pei, Hongtao Yang

**Affiliations:** ^1^Renal Department, First Teaching Hospital of Tianjin University of Traditional Chinese Medicine, 314 Anshan Xi Road, Nan Kai District, Tianjin 300193, China; ^2^Medical Experiment Center, First Teaching Hospital of Tianjin University of Traditional Chinese Medicine, 314 Anshan Xi Road, Nan Kai District, Tianjin 300193, China; ^3^Tianjin Key Laboratory of Translational Research of TCM Prescription and Syndrome, 314 Anshan Xi Road, Nan Kai District, Tianjin 300193, China

## Abstract

**Purpose:**

Hirudin, a polypeptide structure containing 65 amino acids, is a potent natural thrombin inhibitor with anticoagulant property extracted from *Hirudo medicinalis*. It has been reported to have anti-inflammatory and antifibrotic property. Here we explored the renoprotective effect of hirudin on unilateral ureteral obstruction (UUO) induced renal interstitial fibrosis (RIF).

**Methods:**

Rats were randomly divided into five groups: sham group, UUO alone group, and three UUO + hirudin-treatment groups (10, 20, or 40 IU/kg/d, for 14 continuous days). At the end of the experiment period, animals were sacrificed. Pathologic changes in renal specimens were observed using hematoxylin and eosin (HE) staining and Masson staining. The expressions of collagen III (Col III), fibronectin (FN), *α*-smooth muscle actin (*α*-SMA), protease-activated receptor 1 (PAR-1), and proteins in the TGF-*β*1/Smad and NF-*κ*B pathways in renal tissues were examined by immunohistochemistry and/or Western blotting.

**Results:**

HE and Masson staining showed that hirudin-treated UUO rats had lower extent of renal injury and deposition of extracellular matrix (ECM) in renal interstitium than those in the UUO group. The results of immunohistochemistry and WB indicated decreased protein expressions of Col III, FN, *α*-SMA, PAR-1, and inflammatory markers such as tumor necrosis factor-*α* and interleukin-6 after hirudin treatment. Furthermore, hirudin reduced the expressions of transforming growth factor *β*1 (TGF-*β*1), phosphorylated-Smad2, and phosphorylated-Smad3 in the UUO model. In parallel, we found inhibited nuclear factor-*κ*B (NF-*κ*B) signaling after hirudin treatment, with downregulated protein expressions of P65, phosphorylated-P65, and phosphorylated-i*κ*B*α* and increased i*κ*B*α*.

**Conclusion:**

Hirudin improves kidney injury and suppresses inflammatory response and ECM accumulation in UUO rats; its underlying mechanism may be associated with the inhibition of TGF-*β*1/Smad and NF-*κ*B signaling.

## 1. Introduction

Renal interstitial fibrosis (RIF) characterized by the excessive deposition of extracellular matrix (ECM), alteration of tubular structure, and activation of fibroblasts is a common pathological pathway resulting in end-stage renal disease. Transforming growth factor *β*1 (TGF-*β*1)/Smads signaling plays an important role in activating myofibroblasts with excessive extracellular matrix (ECM) production in tubular interstitial [1, 2]. Studies have shown that TGF-*β*1/Smads signaling could induce the transdifferentiation of renal intrinsic and tubular epithelial cells into myofibroblast-like phenotype and function [3–5], as well as recruiting and predisposing bone marrow derived fibrocytes into myofibroblast cells [6, 7]. Apart from this, TGF-*β*1 is crucial in activating subsequent nuclear factor-*κ*B (NF-*κ*B) signaling with a downstream inflammatory effect, including the release of growth factors and proinflammatory cytokines, such as tumor necrosis factor-*α* (TNF-*α*), interleukin-1*β* (IL-1*β*), and monocyte chemotactic protein-1 (MCP-1) [8]. The activation of TGF-*β*1/Smads and NF-*κ*B signaling is thus a precursor which accelerates and amplifies the progress of renal fibrosis. We believe that inhibiting TGF-*β*1/Smads and NF-*κ*B signaling is beneficial in preventing or delaying the progression of RIF, at least in part, by restraining the accumulation of ECM and occurrence of inflammation induced by unilateral ureteral obstruction (UUO).

Hirudin, an extract from salivary glands of medicinal leeches *(Hirudo medicinalis)*, is one of the most potent natural inhibitors of thrombin. The cellular effect of hirudin is, however, more than anticoagulant effect—by forming a 1 : 1 stoichiometric complex with thrombin, hirudin can inhibit thrombin mediated protease-activated receptor 1 (PAR-1) activation cellular transduction [9, 10]. PAR-1, one of the G-protein coupled protease-activated receptors, is widely expressed on glomerular endothelial and tubule cells [11]. Its activation has been reported to be involved in the process of inflammation and fibrosis [12–14]. The role of PAR-1 in RIF has been suggested by Waasdorp et al. who demonstrated that PAR-1 knockdown UUO model could attenuate the accumulation of fibroblasts, macrophages, and relevant fibrosis response (e.g., decreased levels of MCP-1 and TGF-*β*1) [15]. Moreover, Bao et al. showed that hirudin can reduce the release of TNF-*α* and matrix metalloproteinase 12 by inhibiting the activation of PAR-1 in lipopolysaccharide induced lung injury rats [16]. The mechanism was yet not fully explained and, to the best of our knowledge, the effect of hirudin on RIF has not been delineated. Here, we explored the potentially antifibrotic effect of hirudin on RIF in a well-established UUO renal fibrosis rats model and examined components in the TGF-*β* and NF-*κ*B signaling pathway to elucidate the underlying molecular mechanisms.

## 2. Materials and Methods

### 2.1. Animal Model

Sprague–Dawley (SD) male rats (seven to eight weeks old, body weight 200 ± 20 g) were provided by Beijing Vital River Laboratory Animal Technology Co., Ltd. (Beijing, China), and were adaptively housed for one week in a specific-pathogen-free (SPF) environment under a controlled temperature (22 ± 2°C), relative humidity (45 ± 5%), and a 12 h light/dark cycle prior to operation. The study protocol was approved by Animal Research Committee at Tianjin University of Traditional Chinese Medicine and in accordance with the NIH Guide for the Care and Use of Laboratory Animals (2011).

Left unilateral ureteral obstruction was performed using an established protocol as described previously [17]. To evaluate the effect of hirudin (patent no. ZL03113566.8, 100 IU/vial, Canton Xike Kang Biotechnology Co., Ltd., Guangxi, China) on renal fibrosis, the rats were weighed and randomized into five groups (*n* = 30): (a) the sham-operated group with normalized saline by tail vein injection (*n* = 6); (b) the UUO group with normalized saline by tail vein injection (*n* = 6); (c) UUO hirudin treatment group with a dose of 10 IU/kg/d by tail vein injection (*n* = 6); (d) UUO hirudin treatment group with a dose of 20 IU/kg/d by tail vein injection (*n* = 6); (e) UUO hirudin treatment group with a dose of 40  IU/kg/d by tail vein injection (*n* = 6). Hirudin was given shortly after the operation and all rats were sacrificed after 14 consecutive days of treatment. The blood plasma and left ligated kidney samples were collected for biochemistry, histology, immunohistochemistry, and Western blot (WB) analysis.

### 2.2. Serum Biochemical Examination

The serum creatinine (Scr) and alanine aminotransferase (ALT) levels were determined using a Creatinine Assay Kit and an Alanine Aminotransferase Assay Kit (Nanjing Jiancheng Bioengineering Institute, China).

### 2.3. Morphology and Immunohistochemistry Analysis

The left kidney tissues were immobilized in 10% neutral formalin solution for 24 hours and then underwent gradient dehydration, transparency, paraffin immersion, paraffin embedding, and hematoxylin and eosin (HE) staining. To assess histopathologic changes in the kidney, 10 high magnification (×200) was randomly selected in each sample and scoring method was based on Tervaert et al. [18]. For Masson dyeing, the histological sections underwent dewaxing and dehydration; then the tissues were stained with hematoxylin and Ponceau red liquid dye acid complex. The tissues were then directly stained with aniline blue liquid and 1% acetic acid after soaking in 1% phosphomolybdic acid solution. For the analysis of fibrotic area, 10 high magnification (×200) was randomly selected and photographed in each sample. Collagen observed as the blue area represents the extent of the fibrosis, and the areas were calculated by image analysis software (Image-Pro Plus 6.0) for semiquantitative score.

For immunohistochemistry staining, the activity of endogenous enzymes was blocked by 3% hydrogen peroxide after the kidney sections were repaired by microwave antigen for 15 min; then the tissue was blocked by goat serum for 15–20 min. Primary antibodies used in this research contained alpha-smooth muscle actin (*α*-SMA, 1 : 200, Abcam, ab5694), fibronectin (FN, 1 : 100, Proteintech, 15613-1-AP), and PAR-1 (1 : 50, Abcam, ab32611) overnight at 4°C. After washing with phosphate-buffered saline (PBS), the sections were covered with secondary antibody for 1h and then accordingly 3,3′-diaminobenzidine (DAB) and hematoxylin were added. 10 high magnification (×400) was randomly selected in each sample. The percentage of positive staining area was calculated by image analysis software (Image-Pro Plus 6.0) for semiquantitative score.

### 2.4. Western Blot Analysis

Renal tissues were lysed on ice with RIPA lysis buffer (Boster, China) that contained protease inhibitor and phosphatase inhibitor and then centrifuged at 10000 *g* and 4°C for 10 minutes after 30 minutes of static duration. The supernatant was taken and the protein concentration was determined by BCA kit (Boster, China). Samples with equal concentrations of tissue protein (40 *μ*g) were mixed with a 2 × sample buffer, and then they were separated on 10% SDS-polyacrylamide gels (SurePAGE, GenScript). After electrophoresis, the proteins were transferred to polyvinylidene fluoride (PVDF) membrane by wet rotation method; then the membrane was blocked by 5% skimmed milk powder at room temperature for 1 hour. Membranes were incubated at 4°C overnight with anti-FN (1 : 2000, Proteintech, 15613-1-AP), anti-Col III (1 : 1000, Abcam, ab184993), anti-*α*-SMA (1 : 500, Abcam, ab5694), anti-PAR-1 (1 : 200, Abcam, ab32611), anti-TGF-*β*1 (1 : 750, Abcam, ab92486), anti-p-Smad2 (1 : 400, Boster, BM4695), anti-Smad-2 (1 : 400, Boster, BA4557), anti-p-Smad3 (1 : 1000, CST, 9520), anti-Smad3 (1 : 1000, CST, 9513), anti-IL-6 (1 : 500, Proteintech, 21865-1-AP), anti-TNF-*α* (1 : 1000, Abcam, ab6671), anti-p-i*κ*B*α* (1 : 1000, Abcam, ab195751), anti-i*κ*B*α* (1 : 500, Abcam, ab12134), anti-p-P65 (1 : 2000, Abcam, ab86299), anti-P65 (1 : 750, Abcam, ab131485), and anti-GADPH (1 : 10000, Proteintech, 10494-1-AP). After washing with TBS-T, the membranes were incubated with HRP-conjugated AffiniPure goat anti-mouse IgG (1 : 10000, Proteintech, SA00001-1), or anti-rabbit IgG (1 : 10000, Proteintech, SA00001-2) for 1h at room temperature. After washing, the membranes were added with enhanced chemiluminescence system (ECL) detection kit (Boster, China). The positive bands were detected with ChemStudio Imaging System (German) and quantified with VisionWorks program (USA).

### 2.5. Statistical Analysis

Statistical analysis was performed by SPSS software v25.0. Data were presented as mean ± standard deviation (SD). Multiple comparisons were done by one-way analysis of variance (ANOVA) followed by Turkey multiple comparisons test. In the case of nonnormally distributed data, the Kruskal–Wallis test was used. *P* value <0.05 was considered statistically significant.

## 3. Results

### 3.1. Serum Biochemical, Kidney Morphological, and Histological Changes

**Figure 1:**
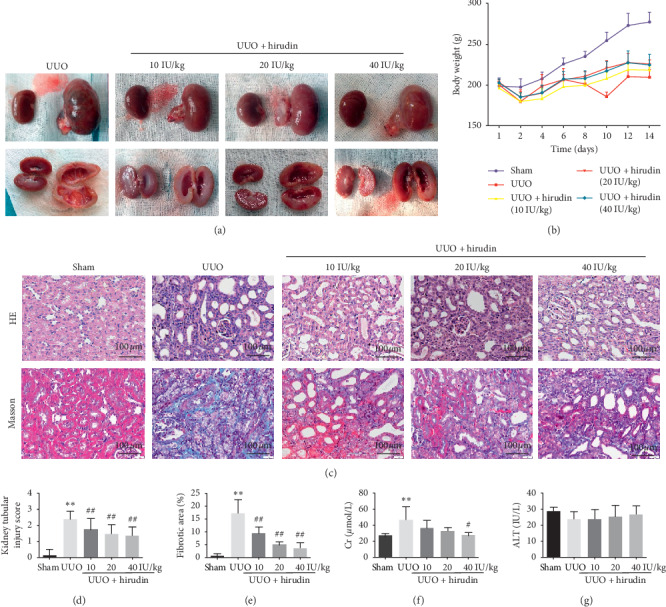
Effect of hirudin on kidney injury, body weight, serum Scr, and ALT level in UUO rats. (a) Kidney tissues collected in different groups. (b) Body weight among five groups. (c) HE (200×) and Masson (200×) staining of the experimental groups. (d) Kidney tubular injury score, based on HE staining. (e) Semiquantitative analysis of average optical density of fibrotic area, based on Masson staining. (f, g) Serum Scr and ALT level. Data are expressed as mean ± SD. For comparison among the UUO group and the control group, ^*∗∗*^indicates *P* < 0.01. For comparison among the hirudin-treated groups and UUO group, ^#^ indicates *P* < 0.05 and ^##^ indicates *P* < 0.01.

Compared with the contralateral kidneys, the size of the obstructed kidneys in UUO group was visually larger and the demarcation between cortex and medulla became unclear; such changes were attenuated after hirudin treatment (Figure 1(a)). In addition, UUO decreased the body weight of animals as compared to sham; no obvious differences were found between UUO group and hirudin treatment groups (Figure 1(b)).

UUO rats were characterized by severe tubular dilatation or atrophy, interstitial fibrosis and inflammatory cell infiltration (HE staining), and substantial deposition of collagens in the renal interstitium (Masson staining), and such histological changes were obviously alleviated in hirudin-treated groups; semiquantitative positive area analysis further confirmed these observations (Figures 1(c)–1(e)).

UUO operation induced apparent increases in Scr level, and hirudin treatment attenuated increased Scr level (Figure 1(f)). There were no significant differences in ALT level after UUO or hirudin treatment (Figure 1(g)).

### 3.2. ECM Accumulation and *α*-SMA Expression in UUO Rats

**Figure 2:**
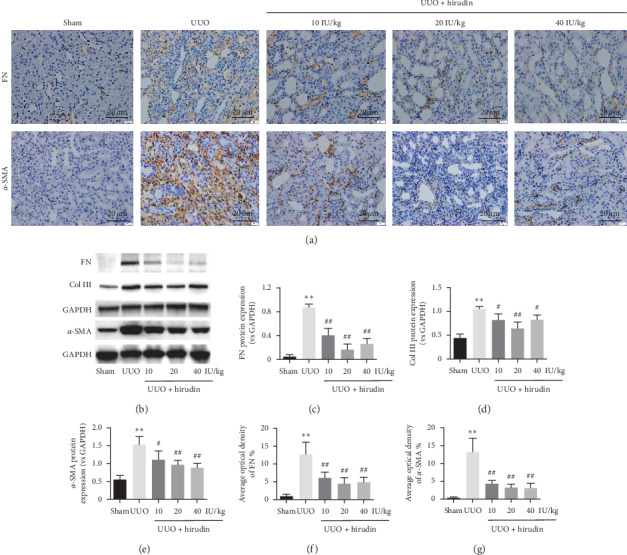
Effect of hirudin on renal interstitial ECM accumulation and activation of myofibroblast in UUO rats. (a) Immunohistochemical staining of FN and *α*-SMA (magnification ×400). (b) Western blot analysis of FN, collagen III, and *α*-SMA; GAPDH was used as the loading control. (c–e) Protein levels of FN, collagen III, and *α*-SMA in each group. (f, g) Average optical density of FN and *α*-SMA. Data are expressed as mean ± SD. For comparison among the UUO group and the control group, ^*∗∗*^indicates *P* < 0.01. For comparison among the hirudin-treated groups and UUO group, ^#^ indicates *P* < 0.05 and ^##^ indicates *P* < 0.01.

In immunohistochemistry analysis, the accumulation of FN and *α*-SMA positive cells distributed in the interstitium was significantly decreased in hirudin treatment groups compared with UUO group (Figures 2(a), 2(f), and 2(g)). In accordance, compared with UUO group, Western blot showed decreased protein expression of FN, collagen III, and *α*-SMA in the three different doses of hirudin groups, respectively (Figures 2(b)–2(e)). Collectively, hirudin can significantly reduce UUO-induced accumulation of ECM by regulating the expression of FN, collagen III, and *α*-SMA.

### 3.3. PAR-1 Expression in UUO Rats

**Figure 3:**
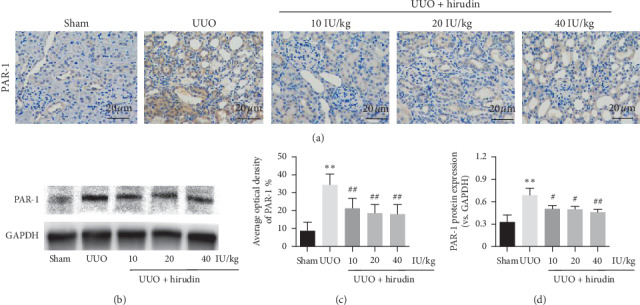
Effect of hirudin on the expression of PAR-1 in UUO rats. (a) Immunohistochemical staining of PAR-1 (magnification ×400). (b) Western blot of PAR-1; GAPDH was used as the loading control. (c) Average optical density of PAR-1. (d) Protein levels of PAR-1. Data are expressed as mean ± SD. For comparison among the UUO group and the control group, ^*∗∗*^indicates *P* < 0.01. For comparison among the hirudin-treated groups and UUO group, ^#^indicates *P* < 0.05 and ^##^ indicates *P* < 0.01.

Immunohistochemical staining results also showed that while PAR-1 positive expression was widely distributed in renal interstitium in UUO group, it was visibly mitigated in hirudin-treated groups. This was further confirmed in semiquantitative positive area analysis and Western blot (Figures 3(a)–3(d)). These results demonstrated that PAR-1 activation was involved in UUO-induced RIF, and hirudin has the inhibitory effect on PAR-1 expression.

### 3.4. TGF-*β*1/Smad Signaling in UUO Rats

**Figure 4:**
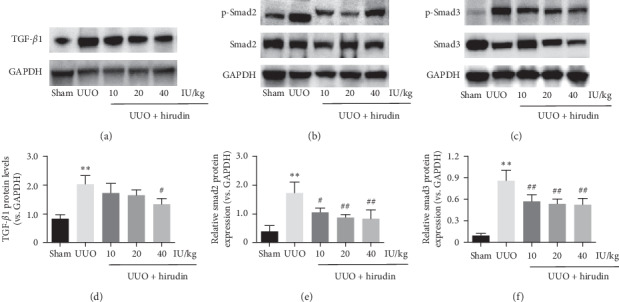
Effect of hirudin on the expression of TGF-*β*1/Smad signaling in UUO rats. (a–c) Western blot of TGF-*β*1, p-Smad2, Smad-2, p-Smad3, and Smad3; GAPDH was used as the loading control. (d–f) Protein levels of TGF-*β*1, p-Smad2/Smad-2, and p-Smad3/Smad-3 in each group. Data are expressed as mean ± SD. For comparison among the UUO group and the control group, ^*∗∗*^indicates *P* < 0.01. For comparison among the hirudin-treated groups and UUO group, ^#^indicates *P* < 0.05 and ^##^ indicates *P* < 0.01.

In accordance with the results above, we found the protein levels of TGF-*β*1 and the ratio of p-smad2/smad2 and p-smad3/smad3 in UUO group are higher than those in sham group, suggesting the activation of TGF-*β*1/Smad signaling. However, hirudin dose dependently reversed UUO-induced expression changes in TGF-*β*1, p-smad2/smad2, and p-smad3/smad3 (Figures 4(a)–4(f)). In summary, hirudin suppressed UUO-induced TGF-*β*1/Smads signaling activation in the left kidneys of UUO rats.

### 3.5. Proinflammatory Cytokines and NF-*κ*B Signaling in UUO Rats

**Figure 5:**
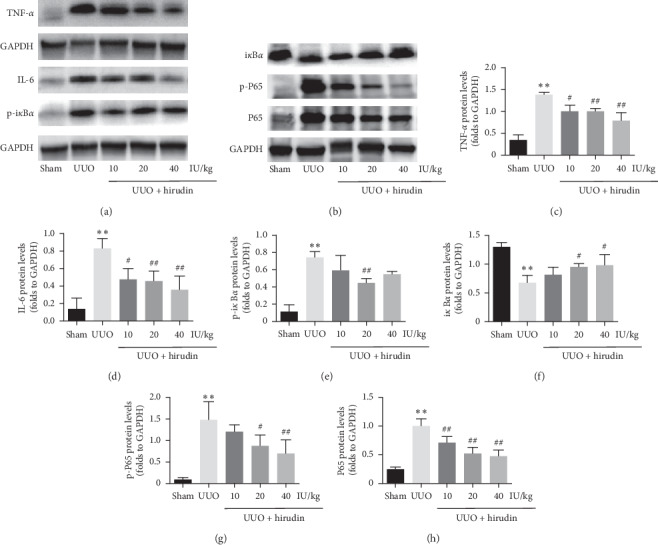
Effect of hirudin on protein expression of IL-6, TNF-*α*, and NF-*κ*B signaling in UUO rats. (a, b) Western blot analysis of IL-6, TNF-*α*, p-i*κ*B*α*, i*κ*B*α*, p-P65, and P65; GAPDH was used as the loading control. (c–h) Protein levels of IL-6, TNF-*α*, p-i*κ*B*α*, i*κ*B*α*, p-P65, and P65 in each group. Data are expressed as mean ± SD. For comparison among the UUO group and the control group, ^*∗∗*^indicates *P* < 0.01. For comparison among the hirudin-treated groups and UUO group, ^#^ indicates *P* < 0.05 and ^##^ indicates *P* < 0.01.

We found that the protein levels of IL-6 and TNF-*α* in UUO group were higher than those in sham group and the levels were reduced in hirudin treatment groups in a dose-dependent manner (Figures 5(a), 5(c), and 5(d)). To elucidate NF-*κ*B signaling in RIF, we detected the protein level of P65 and i*κ*B*α*. Compared with sham group, the protein levels of total P65, p-P65, and p-i*κ*B*α* in UUO group were significantly increased, while the level of i*κ*B*α* was decreased. Hirudin treatment reversed these changes in UUO rats (Figures 5(a), 5(b), 5(e)–5(h)). Taken together, these results indicated that hirudin prevented the NF-*κ*B signaling thereby suppressing the inflammatory response in the left kidneys of UUO rats.

## 4. Discussion

In this current study, we hypothesized that hirudin would mitigate RIF by inhibiting TGF-*β*1/Smads and NF-*κ*B signaling. Thus, we explored the antifibrotic effect of hirudin on RIF in vivo with UUO rats. We found that, compared with UUO group, hirudin treatment significantly mitigated inflammatory infiltration and ECM accumulation in the pathological development of RIF and this was accompanied by downregulated expression of TGF-*β*1/Smads and NF-*κ*B signaling. Our results strongly suggested hirudin was effective in improving RIF by targeting TGF-*β*1/Smads and NF-*κ*B signaling.

The role of PAR-1 in RIF has been suggested by Waasdorp et al. in a PAR-1 knockdown UUO model [15]. In accordance, immunohistochemistry staining and Western blot in our study showed that, compared with sham group, PAR-1 expression was significantly increased in UUO group, while hirudin treatment sufficiently decreased the extent of PAR-1 expression, which was accompanied by downregulated signaling of TGF-*β*1 and NF-*κ*B and consequent improved inflammation and fibrosis in renal interstitium. Consistent with this notion, our results further proved the effectiveness of hirudin on interfering PAR-1-dependent pathway in UUO-induced RIF model.

TGF-*β*1 was a well-known fibrogenic factor which can promote ECM accumulation by activating myofibroblasts and inhibiting matrix degradation [19, 20]. Therefore, blockade of TGF-*β*1 signaling may be an effective strategy for the treatment of RIF and numerous studies have so far suggested that TGF-*β*1 was a promising target in renal antifibrotic therapy [21–23]. In the canonical TGF-*β*1 signaling pathway, TGF-*β*1 binds to its type II receptor (T RII)-kinase and then activates its type I receptor (T RI)-kinase, which would lead to the activation and phosphorylation of downstream substrates of Smad2 and Smad3. Phosphorylated Smad2/3 binds to Smad4 and translocates into the nucleus to regulate the target gene transcription. Recent studies have demonstrated the activation of Smad2/3 in TGF-*β*1 signaling-mediated renal fibrosis [20, 24]. Echinacoside (ECH), a natural polyphenolic compound, has been reported to downregulate *α*-SMA levels and decrease ECM accumulation in diabetic nephropathy model rats through inhibition of the TGF-*β*1/Smad pathway [25]. To explore whether hirudin could suppress activated TGF-*β*1/Smad signaling in UUO rats, we investigated the TGF-*β*1, Smad2, and Smad3 expression in the studied models. Our results showed that, apart from inhibiting PAR-1 expression, hirudin treatment significantly decreased the protein expression of TGF-*β*1 and the ratio of p-Smad2/Smad2 and p-Smad3/Smad3 in UUO rats model. Hence, these findings again proved that hirudin could inhibit UUO-induced fibrosis through TGF-*β*1/Smads signaling activation.

Abundant evidence demonstrated the important role of inflammation in the initiation and progression of RIF [26–29]. IL-6 was an important mediator in renal injury and fibrosis [30, 31]. In this study, HE staining identified vast scale of inflammatory cells infiltrating into the renal interstitium in UUO rat model. Consistent with this result, Western blot data showed UUO rats had higher protein levels of IL-6 and TNF-*α* than those in sham group. In contrast, the expression of these proteins in hirudin treatment groups was significantly diminished. While renal injury would trigger inflammatory response in renal interstitium, inflammation could in turn aggravate the pathological injury and promote fibrosis in kidney [32, 33]. Our results indicated that hirudin treatment could effectively inhibit the secretion of proinflammatory cytokines in UUO rats. More importantly, NF-*κ*B, a key factor of inflammatory signaling, could once be phosphorylated and enter into the nucleus, activating the genes of inflammatory and fibrogenic factors. It has been reported that NF-*κ*B signaling was activated in UUO models, and the blockade of NF-*κ*B signaling would improve renal fibrosis [34, 35]. Our results found significantly elevated protein levels of P65, p-P65, and p-i*κ*B*α* and decreased level of i*κ*B*α* in UUO rats, whereby hirudin efficiently reversed these changes. These results suggested the anti-inflammatory effect of hirudin may be related with the blocking effect on NF-*κ*B signaling transduction.

In summary, our study indicates that hirudin could improve kidney injury and suppress inflammatory response and ECM accumulation in UUO rats; its underlying mechanism may be associated with the inhibition of TGF-*β*1/Smad and NF-*κ*B signaling. Of note, we did not find a dose-dependent trend of the antifibrotic effect of hirudin among the treatment groups in the current experimental set. One possible reason could be the variety of individual sensitivity to the drug as well as a dose deviation during the injection of tail vein. The optimization of effective concentrations of hirudin on RIF in future studies is thus warranted. Moreover, it still deserves to investigate the exact mechanism behind the antifibrotic effect of hirudin and test if PAR-1 acts as the upstream trigger of TGF-*β*1/Smad and NF-*κ*B signaling. This in vivo study, however, indicates a new therapeutic effect for hirudin to prevent renal interstitial fibrosis.

## Data Availability

The data used to support the findings of this study are available from the corresponding author upon request.
